# Supplementary material for: Modified Biopolymer Adsorbents for Column Treatment of Sulfate Species in Saline Aquifers
Source: Materials (Basel). 2020 May 23;13(10):2408. doi: 10.3390/ma13102408 (PMC7288291; doi:10.3390/ma13102408)
Supplement: Supplementary file 1 [file materials-13-02408-s001.pdf]

materials

MDPI

Supplementary Material

Modified Biopolymer Adsorbents for Column Treatment of Sulfate Species in Saline Aquifers

Mostafa Solgi [1], Lope G. Tabil [2] and Lee D. Wilson [1,*]

[1] Department of Chemistry, University of Saskatchewan, 110 Science Place, Saskatoon, Saskatchewan S7N 5C9, Canada; mos023@mail.usask.ca
[2] Department of Chemical and Biological Engineering, University of Saskatchewan, 57 Campus Drive, Saskatoon, Saskatchewan S7N 5A9, Canada; lope.tabil@usask.ca
* Correspondence: lee.wilson@usask.ca; Tel.: +1-306-966-2961; Fax: +1-306-966-4730

Received: 3 May 2020; Accepted: 20 May 2020; Published: 23 May 2020

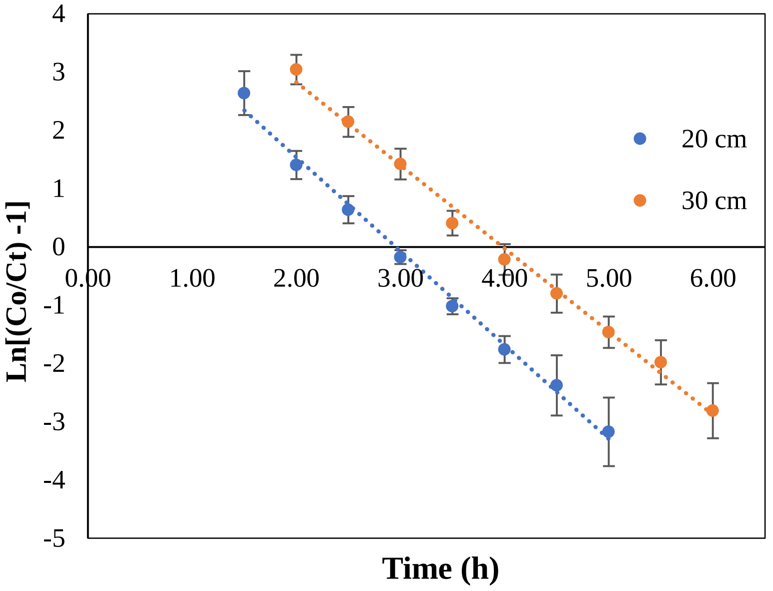

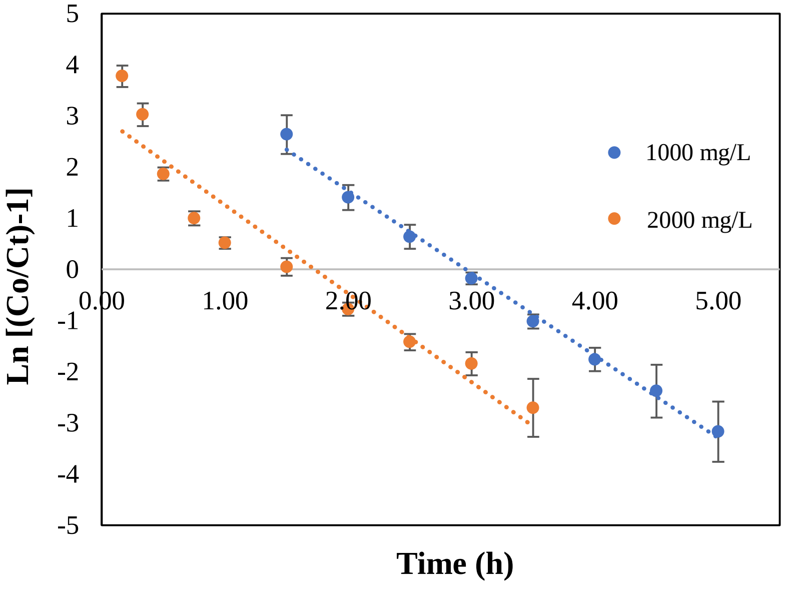

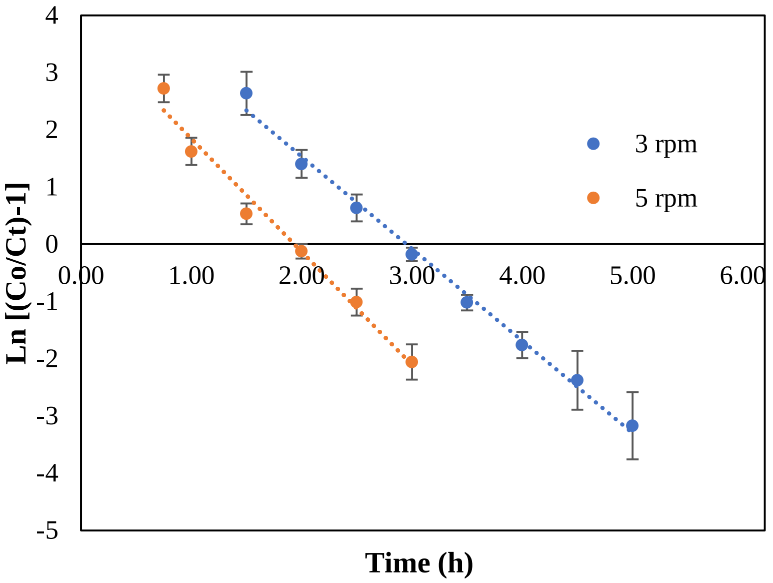

Figure S1. (**a**) bed depth, (**b**) influent concentration, and (**c**) flow rate. Linear plots of Thomas model at different initial sulphate concentration (pH = 4.5, temperature 25 ± 2 °C, bed height 20 cm, flow rate 3 mL/min).

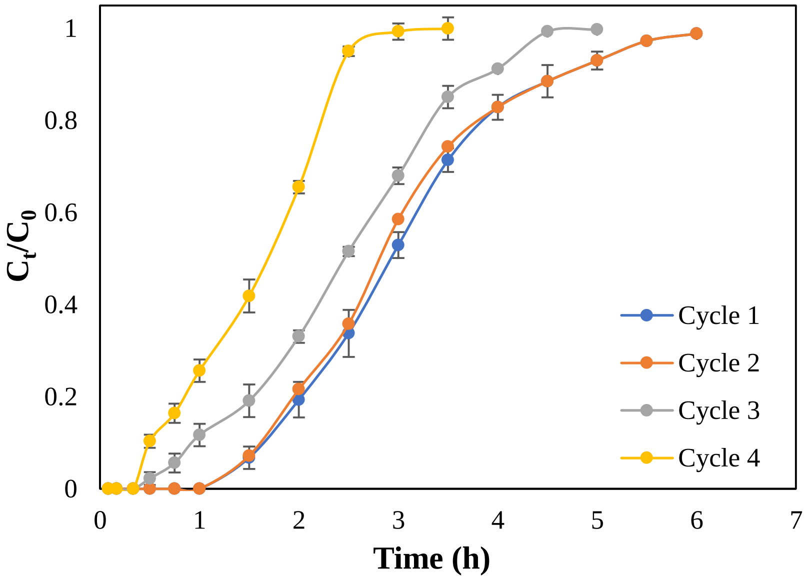

Figure S2. Breakthrough curves of sulphate adsorption by Ca-CP for cycle 1 to cycle 4 (pH 4.5, bed height of 20 cm, flow rate of 3 mL/min, initial sulphate concentration of 1000 mg/L).
